# Anlotinib combined with pembrolizumab as first-line treatment for advanced pulmonary sarcomatoid carcinoma: a case report and literature review

**DOI:** 10.3389/fonc.2023.1241475

**Published:** 2023-10-18

**Authors:** Yingmei Wen, Yi Dong, Lina Yi, Guifang Yang, Mengxia Xiao, Qingqing Li, Chen Zhao, Dafu Ye, Yi Yao

**Affiliations:** ^1^ Cancer Center, Renmin Hospital of Wuhan University, Wuhan, China; ^2^ Department of Pathology, Zhongnan Hospital of Wuhan University, Wuhan, China; ^3^ Department of Oncology, Yichun People’s Hospital, Yichun, China; ^4^ Hubei Provincial Research Center for Precision Medicine of Cancer, Wuhan, China

**Keywords:** pulmonary sarcomatoid carcinoma, anlotinib, pembrolizumab, immune checkpoint inhibitors, genomic analysis, case report

## Abstract

Pulmonary sarcomatoid carcinoma (PSC) is an uncommon variant of non-small cell lung cancer (NSCLC), known for its unfavorable prognosis. Previous studies have elucidated that PSC generally exhibits a significant expression of programmed death-ligand 1 (PD-L1), an elevated tumor mutation burden, and marked vascular invasion. These factors imply the possible effectiveness of treatments like immunotherapy and anti-angiogenic therapy. The subject of this case was a 65-year-old male diagnosed with advanced PSC, characterized by high PD-L1 expression and devoid of known driver gene mutations. Owing to the restrictions imposed by the COVID-19 pandemic, the patient initially underwent home-based treatment with anlotinib, which led to symptomatic improvement after a single treatment cycle. Subsequent hospitalization allowed for the administration of anlotinib plus Pembrolizumab, resulting in a partial response. Radiotherapy was necessitated due to local disease progression. But after 15 cycles of treatment with Pembrolizumab, hyperprogression was observed. The patient’s overall survival spanned 14 months, with no evident adverse reactions to the medications. Genomic analysis revealed potential associations between treatment efficacy and mutations in the *TP53*, *NF1*, and *MET* genes. This case underscores the effectiveness and safety of a first-line treatment regimen combining pan-target anti-angiogenic therapy (anlotinib) with anti-tumor immunotherapy.

## Introduction

Pulmonary sarcomatoid carcinoma (PSC) is a rare, poorly differentiated, highly invasive subtype of non-small cell lung cancer (NSCLC) with a dismal prognosis (1). PSC accounts for 0.1%-0.4% of NSCLC cases, with median survival ranging from 11 to 19 months and a 5-year survival rate between 17% and 29% (2). Currently, there is no specific clinical treatment guideline available for PSC due to its advanced stage at diagnosis and resistance to conventional radiotherapy and chemotherapy; thus novel therapeutic targets or approaches are urgently needed (3, 4). Recent investigations have demonstrated that PSC exhibits high expression levels of programmed death-ligand 1 (PD-L1) (5) as well as an elevated tumor mutation burden (TMB) (6). Furthermore, patients with PSC often exhibit significant blood vessel invasion (BVI) (7), which may indicate a new direction for immunotherapy and anti-angiogenetic therapy in this population.

Pembrolizumab, a highly selective monoclonal antibody targeting PD-1, has been approved by the U.S. Food and Drug Administration for treating advanced malignant melanoma and NSCLC (8). Anlotinib, a multi-target tyrosine kinase inhibitor (TKI), has been approved by the National Medical Products Administration as the third-line therapy in advanced NSCLC. By targeting the vascular endothelial growth factor receptor (VEGFR), fibroblast growth factor receptor (FGFR), platelet-derived growth factor receptor (PDGFR), and c-Kit; anlotinib effectively inhibits tumor angiogenesis and growth (9). We herein report the efficacy of anlotinib combined with pembrolizumab in a patient with advanced PSC.

## Case presentation

**Figure 1:**
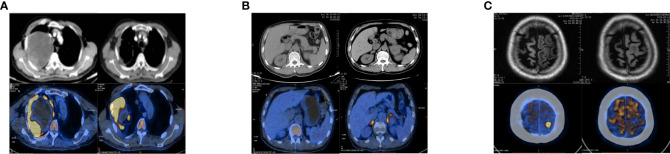
Changes in chest and upper abdomen contrast-enhanced CT, PET-CT, and brain MRI before and after treatment. **(A)** Baseline (left): Chest CT showed a space-occupying mass in the right upper lobe (10.6 × 8.5 cm), and the possibility of lung cancer was considered. PET-CT showed a large soft tissue mass with necrosis in the right upper lobe (12.2 × 11.3 × 12.6 cm) with increased annular metabolism (SUVmax: 27.3). Follow-up after 9 months of treatment (right): Chest CT showed that the lesion in the right upper lung lobe had become smaller (7.8 × 6.0 cm). PET-CT showed that the wall of the nodule and the solid component of the mass in the right upper lobe were smaller than before (6.8 × 9.0 × 7.9 cm), had a slightly lower SUVmax (25.7), involved the right chest wall, and partially extended into the right axilla. **(B)** Baseline (left): Abdominal CT and PET-CT showed no obvious abnormality of the adrenal gland. Nine months after treatment (right): Abdominal CT showed that the left adrenal nodule was enlarged and that a new nodule had formed in the right adrenal gland. PET-CT showed bilateral adrenal soft tissue masses; the larger one was located on the left side and measured about 4.1 × 3.6 cm with an SUVmax of 9.9 to 11.2. **(C)** Baseline (left): Brain MRI showed a left anterior central gyrus nodule (approximately 1.0 cm in diameter), and metastasis was considered. PET-CT showed a slightly dense nodule at the junction of the cortex and medulla in the left frontal lobe (1.1 × 1.4 × 1.0 cm) with an SUVmax of 26.2. Nine months post-treatment (right): Brain MRI showed that the left anterior central gyrus nodule was slightly smaller than the anterior one (about 0.9 cm in diameter), and the post-treatment inhibition of the activity of the metastatic focus was considered. PET-CT showed a slightly dense nodule (diameter of 0.8 cm) at the junction of the cortex and medulla of the left frontal lobe (SUVmax: sparse radioactive distribution). CT, computed tomography; PET-CT, positron emission tomography-computed tomography; MRI, magnetic resonance imaging; SUVmax, maximum standardized uptake value.

A 65-year-old man came to our clinic in April 2020 with complaints of chest pain, persistent coughing for more than 2 months, a progressive right limb weakness for 2 weeks followed by right paralysis for 3 days. Positron emission tomography-computed tomography (PET-CT) revealed a large soft tissue mass in the upper lobe of the right lung, enlarged right pulmonary hilar lymph nodes, and a nodule at the left frontal lobe (**Figures 1A, C**). Brain magnetic resonance imaging (MRI) indicated left anterior central gyrus metastasis (**Figure 1C**). Thus, a clinical diagnosis of stage IV lung cancer (cT4N1M1) was made.

Due to the restrictions imposed by the COVID-19 pandemic, the patient could not undergo definitive pathological diagnosis. Then he was prescribed anlotinib to be taken at home: 12 mg daily for two consecutive weeks followed by a one-week break. Subsequently, notable symptom improvement was observed, with a significant reduction in the size of the lung mass as evident on the CT scan.

**Figure 2:**
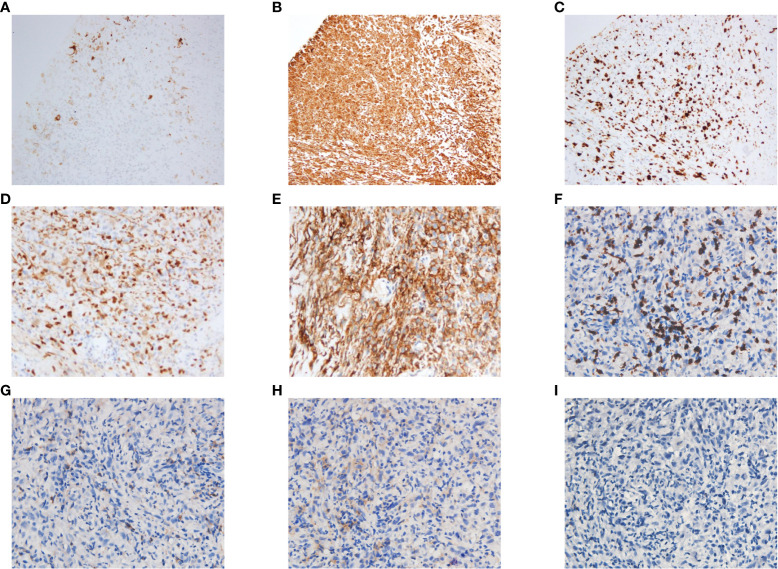
Histopathological staining of the puncture biopsy specimen from the upper lobe of the right lung showed the following results: PCK(−), EMA(focal+), Vim(+), Ki67(+, 60%), S-100(−), CD35(−), CD21(−), CD23(−), CD(scattered+), CD20(−), CK5/6(−), P63(−), CD56(scattered+), Syn(−), P40(−), CK7(−), Napsin A(−), TTF-1(−), ALK(−), AR(focal +), CD30(−), CK8/18(−), Mum-1(−), MyoD1(−), SATB2(+), PD-L1(+, TPS = 80%), CD8(+), CD4(+), CD25(+), Foxp3(−). Some of these results are shown in the figure: **(A)** EMA(focal+). **(B)** Vim(+). **(C)** Ki67(+, 60%). **(D)** SATB2(+). **(E)** PD-L1(+, TPS = 80%). **(F)** CD8(+). **(G)** CD4(+). **(H)** CD25(+). **(I)** Foxp3(−). TPS, tumor proportion score.

**Figure 3:**
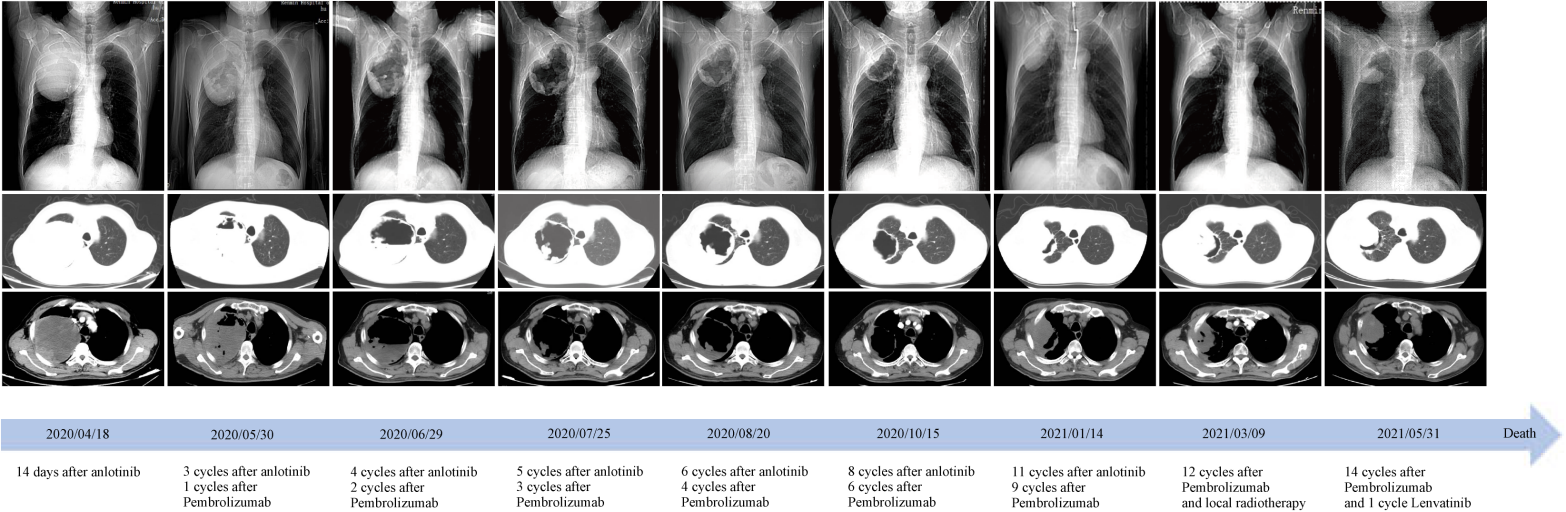
Time course of treatment and changes in the lung CT scans during treatments. Coronal and axial CT scans showed that the central necrotic area of the right upper lobe mass gradually became larger and more cavernous, and the solid cavity wall gradually became thinner. After 9 months of treatment, the right upper lobe mass began to enlarge and invade the chest wall and axilla. CT, computed tomography.

Three weeks later, as the pandemic situation improved in Wuhan, the patient returned to the hospital for biopsies of the right lung lesion. The pathological diagnosis was PSC. Immunohistochemistry results were as follows: EMA (focal+), Ki67 (+, 60%), S-100(−), CK5/6 (−), CD56 (scattered+), Syn (−), P40 (−), napsin A (−), TTF-1 (−), and PD-L1 (+, tumor proportion score = 80%) (**Figure 2**). Next-generation sequencing (NGS) of the tissue sample showed a TMB of 14.46 mutations/Mb, stable microsatellite status, and absence of key driver gene mutations (*EGFR, ALK, BRAF, MET*, etc.) (**Supplementary Table 1**). From then on, the patient underwent anlotinib (12mg ×14d/3 weeks) combined with pembrolizumab (200 mg/3 weeks) for systemic therapy and received radiotherapy for brain metastasis (64 Gy/8 Gy/8 fractions). Regular bi-monthly CT scans of the chest and abdomen showed cavitation necrosis of the tumor expansion and cavity wall thinning (**Figure 3**).

In October, local progression was noted in his adrenal glands, which were treated with radiotherapy (60Gy/2Gy/30 fractions). NGS of peripheral blood sample indicated a TMB of 13.52 mutations/Mb, stable microsatellite status, and an absence of driver gene mutations. After eleven anlotinib cycles and nine pembrolizumab cycles, a January 2021 PET-CT scan indicated systemic progression (**Figures 1A, B**). The patient complained of apparent chest pain near the right axillary and then received palliative radiotherapy for the lesions in right lung and chest wall, followed by the treatment of pembrolizumab solely. However, a deterioration in physical status and systemic tumor hyperprogression was noted after 15 pembrolizumab cycles. Regrettably, the patient passed away in late June 2021, achieving an overall survival (OS) of 14 months.

## Discussion

PSC is typified by its aggressive growth pattern, elevated BVI, and marked resistance to both radiotherapy and chemotherapy. Surgical intervention has been the primary therapeutic approach in early-stage PSC. For advanced, inoperable PSC, first-line treatments predominantly consisted of platinum-based combination chemotherapy. However, the efficacy of chemotherapy for PSC remains limited. A retrospective analysis involving 97 patients revealed that those with advanced PSC who underwent chemotherapy exhibited a median progression-free survival (PFS) of just 2 months and an OS of approximately 6.3 months (3). Addressing PSC therapeutically remains a formidable challenge. Nonetheless, some studies suggest that PSC patients with identifiable driver gene mutations, coupled with high expression of PD-L1, display favorable responses to both molecular targeted therapies and immunotherapies (10, 11). Given the strides in understanding molecular mechanisms pertinent to PSC, this malignancy has entered a new era of targeted therapy and immunotherapy.

Research has increasingly indicated that PSC exhibits characteristics of what are termed “hot tumors”. Compared with other types of NSCLC (such as adenocarcinoma and squamous cell carcinoma), PSC usually demonstrates a higher level of PD-L1 and TMB (12). Concurrently, a significant infiltration of immune cells within the tumor microenvironment (TME) of PSC has been observed, including CD3+ T cells, CD8+ T cells, and macrophages (10, 13). Similar to previous reports (14–16), the pathological evaluation in our case revealed a prominent presence of CD8^+^ T cells, a minimal presence of CD4^+^ T cells, a near absence of T regulator cells, elevated PD-L1 expression and TMB in the tumor tissue (**Figure 2**). These findings allude to the potential efficacy of immune checkpoint inhibitors (ICIs) for such patients. Multiple studies have validated the effectiveness of ICIs in treating PSC (17, 18). Babacan et al. discovered that PD-L1 expression significantly correlated with ICIs efficacy in PSC treatment, with a striking 70.2% of the patients exhibiting high PD-L1 expression achieving either a partial or complete response (11).

Given these findings, we supplemented our patient’s treatment of anlotinib with pembrolizumab post-pathological and molecular-pathological diagnosis. We hypothesize that anlotinib can normalize tumor vasculature and facilitate the entry of tumor-infiltrating lymphocytes, which are reactivated by pembrolizumab, deep into the tumor mass. This may enhance their ability to detect and subsequently target malignant cells (19). Subsequent monitoring showed that with ICIs, there was an increased necrosis in the main lesion in the right lung, leading to tumor tissue liquefaction, cavity formation, and eventual thinning and collapse of the solid cavity wall (**Figure 3**). Although there was local progression in the adrenal glands after 6 months, the patient benefited from the combined systemic treatment of anlotinib and ICIs post-radiotherapy. Overall, the patient’s PFS and OS under this “de-chemotherapy” and dual drug combination as first-line treatment were 6 and 14 months respectively, aligning with survival metrics from other NSCLC clinical studies (14, 19–21).

Immunotherapy has revolutionized the management of NSCLC including PSC. Lee et al. reported that ICIs can markedly enhance PSC prognosis, with a median PFS of 7.2 months and a median OS of 22.2 months (17). Qian et al. conducted a retrospective assessment of ICIs as first-line treatment in 21 PSC patients, identifying median PFS and OS durations of 9.2 and 22.8 months, respectively (22). Their data further highlighted that the PFS and OS outcomes remained consistent across varied immunotherapy strategies, whether it was immunotherapy as monotherapy, in conjunction with anlotinib, or combined with chemotherapy. A phase II study (KCSG-LU16-07) with 18 participants underscored the therapeutic potential and safety of combining durvalumab with tremelimumab for recurrent or metastatic PSCs, documenting a median PFS of 5.9 months and a median OS of 15.4 months (23). When compared to chemotherapy, first-line immunotherapy consistently demonstrates superior outcomes, leading to more pronounced therapeutic results (24). Anlotinib, a multi-target TKI, has shown promise particularly in the management of advanced NSCLC. Both the ALTER0302 and ALTER0303 studies showed that using anlotinib in the third-line treatment or subsequent lines for advanced NSCLC could yield significant PFS improvements (25, 26). A phase Ib clinical trial focusing on the combination of sintilimab and anlotinib as primary therapy for advanced NSCLC reported substantial efficacy with a median PFS of 15.0 months (27). Moreover, anlotinib combined with ICIs demonstrated superior efficacy over anlotinib monotherapy in the third-line treatment of advanced NSCLC, suggesting the synergistic anti-tumoral potential of combining anlotinib with ICIs (28). Dasatinib is another broad-spectrum TKI already approved for the use in patients with NSCLC. Manzotti et al. (29) demonstrated the efficacy of dasatinib in inhibiting the proliferative capacity and attenuating the aggressive properties of PSC *in vitro*, suggesting that dasatinib could potentially emerge as a promising therapeutic avenue for PSC. In this case, due to the constraints posed by the COVID-19 pandemic, the patient initially underwent treatment solely with anlotinib. This treatment alone ameliorated his clinical manifestations, and further enhancements in his condition were observed post the introduction of immunotherapy. Radiological findings also affirmed the patient’s positive response to this combined approach. Current clinical trials (eg. NCT03022500, NCT04725448, NCT04888429, NCT04215913, NCT04224337, NCT02897479), either focusing on mono-immunotherapy or combination strategies for PSC, are underway. We are keenly anticipating the release of their findings.

Molecular pathology plays a pivotal role in the therapeutic strategy of NSCLC. Beyond the frequent mutations in genes like *EGFR* and *ALK*, alterations in *TP53* or *KRAS* can also influence both PFS and OS in patients undergoing immunotherapy (30, 31). Studies in molecular pathology have shown that PSC has mutation profiles akin to high-grade lung adenocarcinoma. This encompasses a high likelihood of *TP53* mutations, as well as alterations in key driver genes like *EGFR* (32). Given PSC’s pronounced mutagenicity, a deeper dive into its genotypic traits may unveil transformative insights (33).

**Figure 4:**
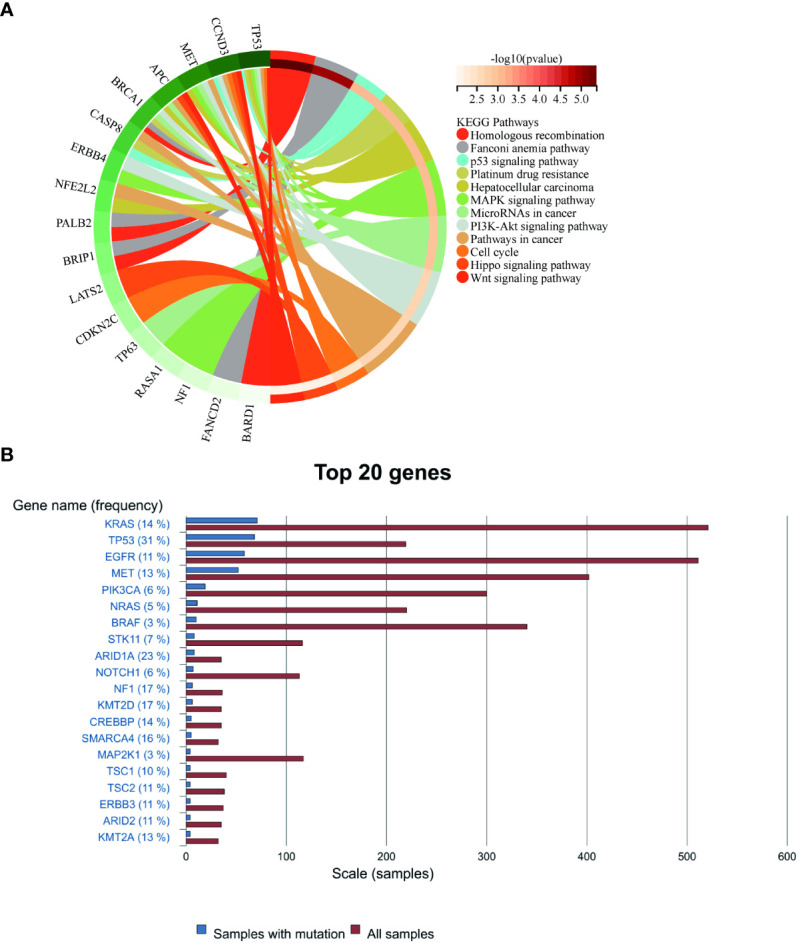
**(A)** Enrichment of mutated genes in the present case. **(B)** The rankings of gene mutation frequencies for PSC from the COSMIC website.

For this purpose, we analyzed the genetic testing results of our patient. Initial assessments revealed an elevated mutation frequency within tumor tissues, particularly noting changes in *NF1, ERBB4*, and *TP53* genes (**Supplementary Table 1**). Post-disease progression, a decline in the prevalence of most gene mutations was observed, a pattern congruent with the findings of Zhou et al (16). Concurrently, Kyoto Encyclopedia of Genes and Genomes (KEGG) analysis of these mutations, both pre- and post-progression, emphasized the regulatory roles of *ERBB4, MET*, and *TP53* within the *MAPK* and *PI3K* signaling pathways. Additionally, *MET* and *NF1* mutations exhibited associations with EGFR-TKI resistance, while *CCND3* modulated the cell cycle (**Figure 4**). Such mutation profiles echo the multiomics research by Yang et al. (15), which highlighted that 57% of subjects exhibited mutations in RTK/RAS pathway genes and 27% had mutations linked to the PI3K and cell cycle pathways. Although no mutation of *EGFR* was detected in this case, the mutations in genes such as *BRCA1*, *NF1*, and *TP53* were discerned (**Supplementary Table 2**). Past research has demonstrated correlations between mutations in *TP53, NF1*, and *MET*, and the efficacy of anti-angiogenetic treatments (34–36). In the latter phase of our patient’s treatment journey, the anticipated “long tail effect” was absent. Three potential causes underpin this observation: first, anlotinib induced downregulation of PD-L1 expression (37). Second, the TME was remodeled due to anti-angiogenic therapy, which consequently hampers anti-tumor immunity (38). Third, the resurgence of tumor hypoxia over time could elevate PD-L1 expression on tumor or stromal cells, facilitating evasion from anti-tumor immune responses (39). Therefore, in clinical settings, meticulous consideration of patient selection, drug dosage, sequencing, combination, and administration timing becomes imperative (40).

The biological characteristics of PSC demonstrate its resistance to conventional chemotherapy, as further reconfirmed by the KEGG results in this case. Therefore, for patients with advanced PSC, targeted treatment or immunotherapy should be considered based on gene detection results (41). In case where a skipping mutation is present in exon 14 of the *MET* gene (*METex14*), capmatinib (10, 42) may be considered as a potential option. If there is a high expression of PD-1/PD-L1 and a high BVI, ICIs can be taken into consideration (43). Hence, building upon this case and previous research findings, we propose that combining first-line anti-angiogenesis therapy with ICIs might represent an effective strategy to enhance treatment response in patients with PSC while avoiding chemotherapy administration. This approach has the potential to significantly improve quality of life and prolong survival time.

## Data availability statement

The datasets presented in this study can be found in online repositories. The names of the repository/repositories and accession number(s) can be found in the article/**Supplementary Material**.

## Ethics statement

The studies involving humans were approved by Clinical Research Ethics Committee of Renmin Hospital of Wuhan University. The studies were conducted in accordance with the local legislation and institutional requirements. The participants provided their written informed consent to participate in this study. Written informed consent was obtained from the individual(s) for the publication of any potentially identifiable images or data included in this article.

## Author contributions

YW and LY collected the data and drafted the manuscript. YD, GY, MX, and LY provided the figures and pathology review and revised the manuscript. QL, CZ, and DY revised the manuscript. YY conceived, organized, performed, reviewed and critiqued the manuscript. All authors contributed to the article and approved the version submitted.
