# Supplementary material for: Anlotinib combined with pembrolizumab as first-line treatment for advanced pulmonary sarcomatoid carcinoma: a case report and literature review
Source: Front Oncol. 2023 Oct 18;13:1241475. doi: 10.3389/fonc.2023.1241475 (PMC10618617; doi:10.3389/fonc.2023.1241475)
Supplement: Supplementary file 1 [file DataSheet_1.pdf]

Supplementary Table 1. Gene mutation status pre- and post-progression in this case

Gene	Mutation site	Amino acid	Frequency1	Frequency2
APC	c.2510G>A	p.S837*	2.00%	-
ATM	c.1236G>A	p.W412*	1.67%	-
ATRX	c.3633T>G	p.D1211E	2.47%	-
BARD1	c.1145A>G	p.N382S	5.73%	-
BRCA1	c.1386delG	p.T464Pfs*11	13.97%	2.94%
BRIP1	c.454G>C	p.D152H	3.55%	-
BRIP1	c.455G>C	p.D153H	3.60%	-
CASP8	c.1065G>C	p.L355F	2.02%	-
CCND3	c.782C>G	p.T261S	15.49%	1.92%
CDKN2C	c.82A>G	p.N28D	17.75%	2.17%
EPHA7	c.475G>C	p.D159H	2.01%	-
ERBB4	c.3361G>T	p.E1121*	23.62%	1.97%
FAM46C	c234C>A	p.H78Q	-	1.46%
FANCD2	c.59CC>T	p.A20V	-	0.41%
HMCN1	c.13573A>G	p.I4525V	8.78%	2.11%
HMCN1	c.4286A>C	p.Y1429S	2.82%	-
INPP4A	c.875G>A	p.R292H	-	0.50%
LATS2	c.490C>T	p.P164S	-	0.43%
MET	c.1990C>G	p.P664A	2.01%	-
NCOR1	c.298G>A	p.D100N	3.82%	-
NCOR1	c.4330G>A	p.V1444M	-	0.42%
NF1	c.4514+1G>A	-	33.25%	3.74%
NFE2L2	c.61_79del19	p.D21Ifs*2	11.40%	-
NFE2L2	c.83_84insC	p.D29Rfs*10	11.27%	-
NLRP7	c.209C>T	p.A70V	-	0.42%
NPM1	c.170T>C	p.L57S	37.84%	2.28%
PALB2	c.398G>C	p.S133T	3.89%	-
POLE	c.1658G>T	p.R553L	12.03%	3.01%
RASA1	c.1254-1G>A	-	13.89%	1.90%
TBX3	c.658-1G>A	-	-	0.42%
TET1	c.5656G>T	p.G1886*	25.84%	2.71%
TGFBR1	c.704C>T	p.S235F	2.57%	-
TMTC4	c.1850A>G	p.N617S	-	0.41%
TP53	c.524G>A	p.R175H	26.97%	3.58%
TP63	c.927C>G	p.N309K	13.15%	2.87%

Supplementary Table 2. Details of genes with clinical significance

Gene	Mutation Site	Nucleotide	Amino Acid	SIFT	Protein Damage	Clinical
BRCA1	41246161	c.1386delG	p.T464Pfs*11	–	–	Pathogenic
NF1	29587534	c.4514+1G>A	–	–	–	Pathogenic
TP53	7578406	c.524G>A	p.R175H	D	D	Pathogenic
PALB2	23647469	c.398G>C	p.S133T	T	B	Controversial
TGFBR1	101900270	c.704C>T	p.S235F	–	D	Unknown
MET	116397716	c.1990C>G	p.P664A	D	D	–
FAM46C	118165724	c.234C>A	p.H78Q	D	D	–
NPM1	170818340	c.170T>C	p.L57S	D	D	–
POLE	133249241	c.1658G>T	p.R553L	D	D	–
EPHA7	94120576	c.475G>C	p.D159H	D	D	–
